# Expressions and Clinical Significance of Met and YAP in Gastric Cancer Tissue Microarray

**DOI:** 10.1155/2024/5591298

**Published:** 2024-04-09

**Authors:** Jinxia Li, Xinyun Zhang, Ying Liu, Jinyong Zhou, Li Shen, Guangxin Yue

**Affiliations:** ^1^Hunan University of Chinese Medicine, Changsha 410208, China; ^2^Provincial Key Laboratory of TCM Diagnostics, Hunan University of Chinese Medicine, Changsha 410208, China; ^3^Institute of Chinese Materia Medica, China Academy of Chinese Medical Sciences, Beijing 100700, China; ^4^Central Laboratory, Affiliated Hospital of Nanjing University of Chinese Medicine, Jiangsu Province Hospital of Chinese Medicine, Nanjing 210029, China; ^5^Institute of Basic Theory of TCM, China Academy of Chinese Medical Sciences, Beijing 100700, China

## Abstract

**Objective:**

This study is aimed at investigating the expression of Met and YAP in gastric cancer and their impact on clinical prognosis.

**Methods:**

Tissue samples and clinical data were collected from 89 patients with gastric cancer. Immunohistochemistry was performed to quantify the expression of Met and YAP using tissue microarray. The correlation between the expressions of Met, YAP, and clinicopathological characteristics of patients was determined using a chi-square test. Survival analysis was conducted using the Kaplan-Meier method, while multivariate survival analysis was performed using the Cox proportional hazard model. Bioinformatics analysis was carried out by downloading chip data from TCGA.

**Results:**

The expression levels of both Met and YAP were significantly higher in gastric cancer tissues compared to adjacent tissues (*P* < 0.001). Met expression showed a positive association with P53 and CD133, whereas YAP expression correlated positively with tumor grade and CD133 (*P* < 0.05). Pearson's analysis revealed a significant correlation between Met expression and VEGFR as well as CD133, while YAP expression correlated with Ki67 and VEGFR (*P* < 0.05). Patients with high levels of both Met and YAP exhibited decreased survival time (*P* < 0.01). Furthermore, Met expression, N stage, and VEGFR were identified as independent risk factors for gastric cancer prognosis (*P* < 0.05), whereas no such association was observed for YAP expression. Bioinformatics analysis demonstrated a significant correlation between the expressions of Met and YAP; both proteins were highly expressed in gastric cancer patients accompanied by markedly reduced survival time.

**Conclusion:**

The expressions of Met and YAP are closely associated with the survival outcomes as well as clinicopathological features in patients with gastric cancer. Moreover, our findings highlight that Met serves as an independent prognostic factor for gastric cancer.

## 1. Introduction

Gastric cancer is a significant global health concern, with China experiencing high incidence and mortality rates, ranking second among all malignant tumors [1, 2]. Surgery is the primary treatment for gastric cancer, while postoperative chemotherapy and radiotherapy can improve survival rates in the short term. However, after five years, only 20-30% of patients survive due to the lack of specific targets and drugs [3]. In recent years, molecular-targeted drugs have shown promising results in treating various types of tumors. Therefore, conducting in-depth research on the pathogenesis of gastric cancer's malignant transformation, metastasis, and invasion mechanisms and identifying specific therapeutic targets are crucial for both basic research and improving clinical efficacy.

One such potential target is the receptor tyrosine kinase c-Met. It predominantly exists on epithelial cell surfaces with tyrosine kinase activity and plays a regulatory role in cellular processes like proliferation and differentiation. Studies have revealed that c-Met is overexpressed in breast cancer, glioma, and other malignancies where its overexpression correlates closely with tumor metastasis [4–7]. Researchers have also found that advanced gastric cancer patients exhibit higher levels of c-Met expression associated with worse prognosis as well as tumor invasion/metastasis status along with tissue differentiation and TNM stage [8–10]. Another key molecule worth investigating is the transcription coactivator YAP/TAZ from the Hippo signaling pathway [11], which functions in growth regulation, development processes, and DNA repair [12]. YAP/TAZ activates gene transcription including SOX4 to promote the maintenance of stemness characteristics within cancer stem-like cells (CSLCs). Overexpression has been observed in breast cancer [13], colorectal cancer [14], gastric cancer [15, 16], and several other malignancies.

Our previous studies have demonstrated that in gastric cancer cells, the binding of c-Met to its ligand HGF leads to a decrease in the levels of phosphorylated Mst1/2, LATS1/2, and YAP/TAZ. Additionally, YAP/TAZ is transported into the nucleus. Further investigations revealed that stimulation with HGF did not significantly enhance the self-renewal ability of gastric cancer cells through the TAZ gene. Therefore, it is likely that c-Met promotes downstream YAP/TAZ transcription, which plays a crucial role in gastric cancer development.

In this study, we aimed to investigate the expressions of c-Met and YAP in gastric cancer tissue microarrays using immunohistochemistry. We also analyzed the correlation between biomarker genes and clinicopathological characteristics in gastric cancer as well as explored the interaction between c-Met and YAP. Through these analyses, we aim to uncover potential mechanisms underlying the involvement of c-Met and YAP in gastric cancer initiation and progression.

## 2. Materials and Methods

### 2.1. Sample Collection

From July 2016 to April 2017, we collected both gastric cancer tissue samples and clinical data from patients at the First Affiliated Hospital of Zhejiang University of Traditional Chinese Medicine. These patients were confirmed to have gastric cancer based on pathology reports and had not undergone radiotherapy, chemotherapy, or immunotherapy treatments. Patients with other malignant tumors besides gastric cancer or those with incomplete or incorrect clinical data were excluded from our study.

As a result, we included a total of 89 patients, comprising 59 males and 30 females, with ages ranging from 32 to 84 years (median age: 65 years). The pathological grading revealed that there were 14 cases classified as grade I-II and 75 cases classified as grade III-IV. Regarding the T stage, there were 12 cases at stage II and 76 cases at stages III-IV. In terms of the N stage, there were 22 cases at stage N0, followed by 14 cases at stage N1, then 23 cases at stage N2, and finally, there were also 30 cases at stage N3. Postoperative pathological TNM staging showed that there were 33 cases at stage I-II and 56 cases at stage III–IV.

### 2.2. Reagents

EnVision™ FLEX+, mouse, high pH, (Link) immunohistochemistry kit (Dako North America company, K8002); antibody diluent (Dako North America company, s3022); Mayer's hematoxylin staining solution (Sigma Aldrich company, SLBT4555); Met (DLC2) XP rabbit mAb (Cell Signaling company, #8198); YAP (D8H1X) XP rabbit mAb (#14074; Cell Signaling Technology).

### 2.3. Instruments

PH-070A thermostatic drying oven (Shanghai Yiheng Scientific Instruments Co., Ltd.), Autostainer Link 48 (Dako North America, Inc.); 1-14 centrifuge (Sartorius), PT Link immunohistochemical pretreatment system (Dako North America, Inc.), ST5010 fully automated stainer instrument (LEICA), and Aperio XT scanner (LEICA).

### 2.4. Chip Fabrication

The collected specimens were stained with hematoxylin and eosin for morphological observations by pathological experts who identified representative lesions of gastric cancer along with adjacent tissues. A tissue microarray instrument (Beecher Instruments, USA) was used to puncture selected areas on wax blocks (aperture size: 1.5 mm; length: 3 mm), which were then implanted into blank wax blocks forming a chip array labeled as No.HStmA180Su15. The resulting arrays were serially sectioned to 4 *μ*m and affixed to 1% polylysine-treated slides.

### 2.5. Immunohistochemistry Detection of Met and YAP

The tissue microarrays were subjected to immunohistochemical analysis. The arrays were dried at 63°C and then hydrated using xylene and gradient alcohol. Antigen retrieval was performed using a DAKO automatic immunohistochemical pretreatment instrument. Slides were rinsed thrice with PBST (phosphate buffered saline with Tween-20) and blocked with 10% goat serum at 37°C for 30 min. Next, the corresponding primary antibodies (Met at a dilution of 1 : 100 and YAP at a dilution of 1 : 500) were added to the slides, which were incubated overnight at 4°C. After rewarming the slides to room temperature, they were cleaned with PBST and placed on a Dako automatic immunohistochemical staining instrument. The Polink-1 HRP DAB detection system was performed at 37°C for 1 h followed by DAB coloration. To visualize cell nuclei, hematoxylin staining was performed for 1 min followed by decolorization using 0.25% hydrochloric acid alcohol solution. After drying, the slides were sealed for further analysis. Aperio XT scanner (LEICA) was used to scan the slides.

### 2.6. Result Determination

Both Met and YAP were localized in the cytoplasm, exhibiting a brown-yellow positive staining signal. Two visual fields were randomly selected for each case, and a total of 200 cells were counted. The staining intensity (0/1+/2+/3+) and positivity rate of Met and YAP were evaluated by two experienced pathologists using a blind method. A semiquantitative scoring system was employed to assess the values as follows: Staining intensity was scored as 0 (negative), 1 (1+), 2 (2+), or 3 (3+); staining positivity score was 0 (negative), 1 (1-25%), 2 (26%-50%), 3 (51-75%), and 4 (76%-100%). The total score was calculated as total score = staining intensity score × staining positivity score. Samples with a total score < 8 were classified into the low expression group, while those with a total score ≥ 8 belonged to the high expression group.

### 2.7. Statistical Analysis

Statistical analysis was performed using SPSS version 23.0 software. The *χ*2 test was utilized to analyze the correlation between Met and YAP expression levels and clinicopathological features. Survival data were analyzed using the Kaplan-Meier method, followed by comparison using the log-rank test. Multifactor survival analysis was conducted using the Cox proportional risk model, while Pearson's test assessed correlation analysis between variables. Statistical significance was set at *P* < 0.05.

### 2.8. Bioinformatics Analysis

Met and YAP expression data were collected from the UCSC (http://genome.ucsc.edu/) database, while human genome annotation information was obtained from NCBI (https://www.ncbi.nlm.nih.gov/). Perl scripting language was used to annotate the gene names. The RStudio software version 3.5.3 was employed for data analysis, with the limma package utilized to extract YAP and MET expression levels. The Ggpubr package was employed for analyzing the differential gene expression between normal and tumor groups, and a thermograph was generated accordingly.

To investigate the correlation of genes with survival in gastric cancer patients, gene expression profiles and clinical information were downloaded from TCGA (https://www.cancer.gov/ccg/research/genome-sequencing/tcga) [17] and KMPLOT (https://kmplot.com/analysis/index.php?p=service&cancer=gastric). Additionally, Gene Expression Profiling Interactive Analysis (GEPIA) (http://gepia.cancer-pku.cn/index.html) [18] was used to confirm the association between Met/YAP expressions and clinicopathologic features in gastric cancer.

Correlation coefficients including Spearman's rank correlation coefficient, Pearson's correlation coefficient, and Kendall's were calculated to analyze the relationships between Met/YAP expressions. Furthermore, TIMER was employed to explore coexpression patterns between YAP and MET.

## 3. Results

### 3.1. Met and YAP Highly Expressed and Correlated in Gastric Cancer

**Figure 1:**
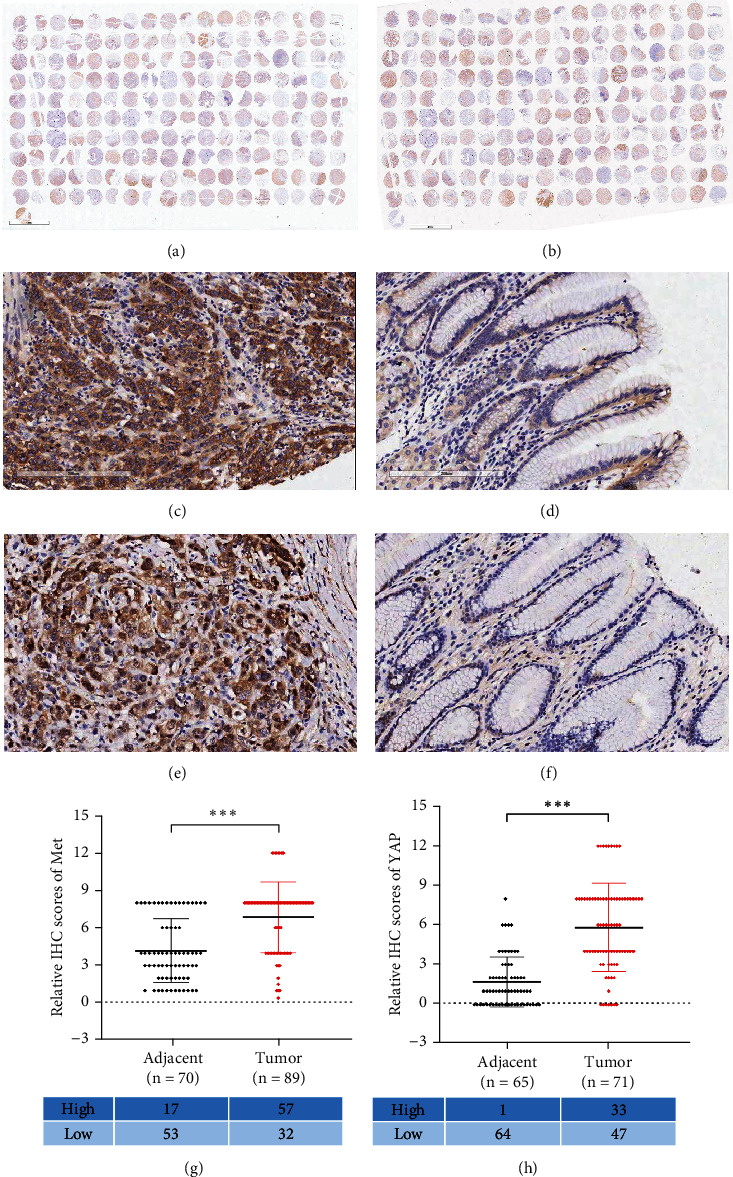
Met and YAP were highly expressed in gastric cancer tissues (IHC). Tissue samples were collected from 89 gastric cancer patients, stained, and semiquantified the Met and YAP expressions by immunohistochemical staining method. (a) Met staining result in tissue microarray; (b) YAP staining result in tissue microarray; (c) Met expression in gastric cancer (×200); (d) Met expression in paracancerous tissues (×200); (e) YAP expression in gastric cancer (×200); (f) YAP expression in paracancerous tissues (×200); (g) relative IHC scores of Met in adjacent and tumor tissues (^∗∗∗^*P* < 0.001); (h) relative IHC scores of YAP in adjacent and tumor tissues (^∗∗∗^*P* < 0.001).

Immunohistochemical detection revealed significantly increased expressions of both Met and YAP in gastric cancer tissues compared to adjacent tissues (*P* < 0.001), as shown in Figure 1.

Furthermore, Pearson's correlation analysis demonstrated a significant positive correlation between Met and YAP expressions (*R* = 0.529, *P* < 0.01).

### 3.2. Correlation Analysis of Met and YAP Expressions with Clinicopathological Features of Gastric Cancer

As shown in Table 1, the *χ*2 test revealed that high expression of Met was positively associated with the expression of P53 and CD133 (*P* < 0.05), but not with age, gender, or tumor stage (*P* > 0.05). On the other hand, high expression of YAP was found to be positively related to the tumor pathological grade and the expression of CD133. Specifically, grade III-IV gastric cancer exhibited higher YAP expression compared to grade I-II gastric cancer (*P* < 0.05). No correlation was observed between sex, age, and YAP expression (*P* > 0.05).

### 3.3. Correlation Analysis of Met and YAP with Tumor-Associated Proteins

Further analysis using Pearson's method demonstrated a significant relationship between Met expression and VEGFR as well as CD133 expressions (*P* < 0.01). Additionally, YAP protein expression correlated with Ki67 and VEGFR protein expressions (*P* < 0.05), as shown in Table 2.

### 3.4. Met and YAP Expressions Negatively Correlated with Overall Survival

**Figure 2:**
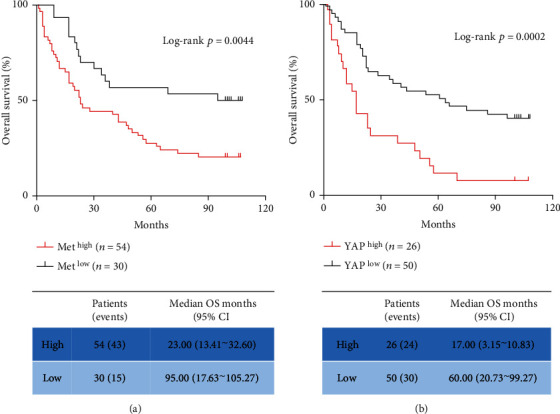
Met and YAP expressions negatively correlated with overall survival. Tissue samples and clinical data were collected from 89 gastric cancer patients, Met and YAP expressions were detected by immunohistochemical staining method, and the Kaplan-Meier survival analysis and log-rank statistical tests were used to analyze the correlation between Met, YAP expressions, and overall survival of patients.

The Kaplan-Meier survival analysis and log-rank tests presented in Figure 2 indicated that patients with high levels of Met and YAP expressions had significantly reduced survival times (*P* < 0.01).

### 3.5. Cox Multivariate Regression Analysis

Univariate analysis revealed that both Met expression, YAP expression, pathological grade, T stage, N stage TNM stage age, and VEGFR expression influenced the prognosis of gastric cancer (*P* < 0.05). Subsequently, the Cox multifactor survival regression analysis showed that Met expression, N stage, and VEGFR expression were independent risk factors for gastric cancer (*P* < 0.05), while YAP expression was not (*P* > 0.05), as displayed in Table 3.

### 3.6. Met and YAP Gene Expression Analysis from the Public Databases

**Figure 3:**
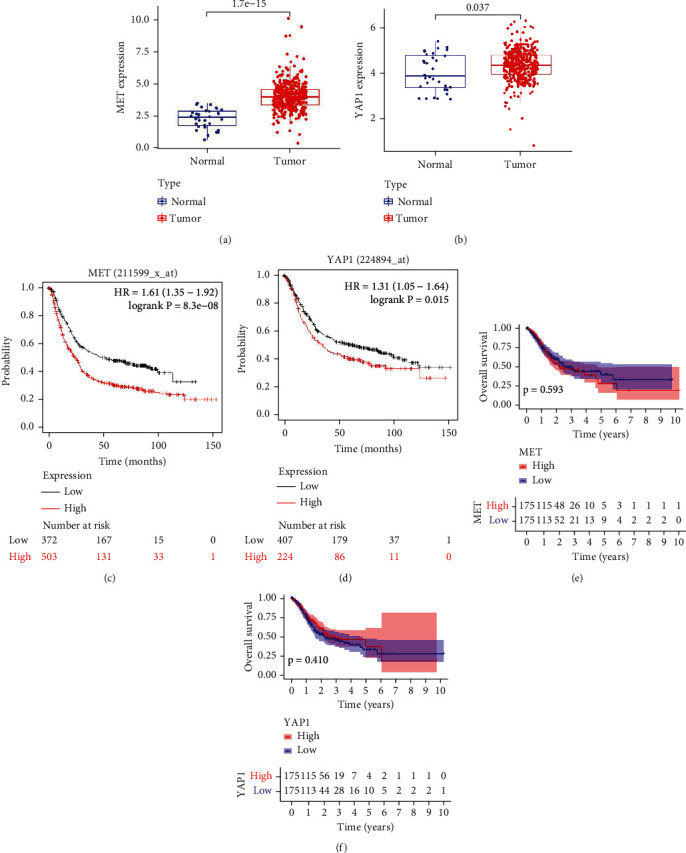
Met and YAP gene expression analysis from the public databases. Bioinformatics analysis was performed by downloading gastric cancer chip data from TCGA. Statistical approaches were employed for survival and correlation analyses. (a) Expressions of Met in gastric cancer chips; (b) expressions of YAP in gastric cancer chips; (c) Met survival analysis by KMPLOT; (d) YAP survival analysis by KMPLOT; (e) Met survival analysis by R package; (f) YAP survival analysis by R package.

We further analyzed the expression and correlation between Met and YAP using the public gene database. The TCGA database provided clinical and gene expression data on 441 gastric cancer cases. As shown in Figures 3(a) and 3(b), increased expression of Met and YAP was significantly associated with different disease states (tumor or normal) (*P* < 0.05). Both Met and YAP expressions in tumor samples were noticeably higher than in normal samples, which is consistent with our previous immunohistochemical results.

To evaluate the correlation between gene expression and overall survival in gastric cancer, we performed KMPLOT analysis. For Met, a total of 875 cases were included with a cutoff value of 210. As shown in Figure 3(c), patients with high expression of Met exhibited shorter survival time (*P* < 0.01), with median survival times of 23.6 months for the high-expression cohort and 51.8 months for the low-expression cohort. For YAP, a total of 631 cases were included with a cutoff value of 3036. As shown in Figure 3(d), high expression of YAP also correlated with worse survival outcomes (*P* < 0.01), with median survival times of 35.9 months for the high-expression cohort and 63.7 months for the low-expression cohort. Figures 3(e) and 3(f) show the same result by using R package.

**Figure 4:**
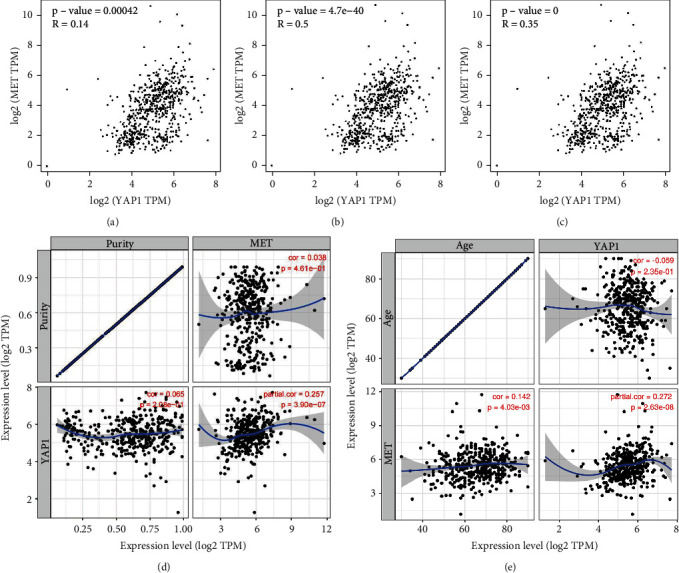
Met and YAP gene correlation analysis from the public databases. Bioinformatics analysis was performed by downloading gastric cancer chip data from TCGA. Statistical approaches were employed for correlation analyses. (a) Correlation between Met and YAP by Spearman's analysis; (b) correlation between Met and YAP by Pearson's analysis; (c) correlation between Met and YAP by Kendall's methods; (d, e) correlation between Met and YAP by the TIMER analysis.

We further assessed the correlation between Met and YAP using Spearman's rank correlation coefficient, Pearson's correlation coefficient, and Kendall's method as depicted in Figures 4(a)–4(c); all three methods showed strong binding between Met and YAP (*R* = 0.14, *P* < 0.001; *R* = 0.50, *P* = 4.7*e*‐40; *R* = 0.35, *P* < 0.001, respectively). Additionally, TIMER analysis revealed that Met was positively correlated with tumor purity and age as illustrated in Figures 4(d) and 4(e). YAP was positively related to tumor purity but negatively related to age. After adjusting for confounding factors such as tumor purity and age, Met and YAP still showed strong correlation (*R* = 0.257, *P* = 3.9*e*‐07; *R* = 0.272, *P* = 2.63*e*‐08, respectively).

## 4. Discussion

Cancer stem-like cells (CSLCs) possess stem-like characteristics in tumor tissues, including self-renewal, differentiation, and high-efficiency tumorigenesis. CSLCs have been identified in various types of tumors such as liver cancer, esophageal cancer, and colorectal cancer [19]. The regulatory mechanisms of CSLCs have been gradually revealed in these tumors. However, the specific signaling molecules and regulatory pathways that maintain the stemness of CSLCs in gastric cancer remain unclear. Therefore, identifying key signaling molecules responsible for maintaining the stemness of gastric cancer CSLCs could provide new insights and methods for targeted therapy.

One potential candidate is c-Met protein which plays a crucial role in maintaining stemness in breast cancer and glioma [5, 6]. High expression of c-Met has also been closely associated with tumor metastasis [7]. In gastric tumors, MET amplification has been reported in approximately 4-10% of patients [20] while overexpression of c-Met protein is observed in 50% of advanced gastric cancers [21, 22]. Studies have shown significantly higher expression levels of c-Met in gastric cancer tissue compared to normal gastric tissue. Furthermore, positive expression of c-Met has been linked to lower efficacy of chemotherapy compared to negative expression cases. Elevated levels of c-Met can activate multiple signaling pathways through autocrine or paracrine signaling mechanisms leading to tumor initiation and progression [23]. In conclusion, understanding the role played by key signaling molecules like c-Met protein in maintaining the stemness properties of CSLCs could pave the way for novel targeted therapies against gastric cancer.

Interestingly, our previous studies have found that c-Met promotes activation of YAP/TAZ in gastric cancer cells which may potentially influence the maintenance of stemness in gastric cancer CSLCs [24]. YAP/TAZ transcription factors, comprising protein kinase in the Hippo pathway, play a crucial role in maintaining cell stemness [25]. YAP/TAZ acts as a transcriptional coactivator of the Hippo signaling pathway [26]. Upon phosphorylation by Mst1/2 and Lats1/2, YAP/TAZ dephosphorylates and dissociates from cytoplasmic 14-3-3 kinase to enter the nucleus where it interacts with TEAD and other factors to initiate transcriptional expression of downstream target genes and promote stemness maintenance in tumor CSLCs [13]. High expression of YAP/TAZ has been reported to promote CSLC stemness maintenance in breast cancer, liver cancer, and melanoma [14–17]. Moreover, studies have shown that HGF/c-Met can regulate the YAP pathway thereby promoting proliferation and migration of tumor cells leading to stemness maintenance of CSLCs in hepatocellular carcinoma and prostate cancer [27, 28]. However, whether c-Met accompanied by YAP/TAZ regulates stemness maintenance of gastric cancer CSLCs remains unclear.

Therefore, this study utilized tissue samples and bioinformatics analysis to investigate the expression of Met and YAP in gastric cancer tissues and analyze their correlation, aiming to elucidate whether the joint effect of Met and YAP contributes to the incidence and prognosis of gastric cancer. Specifically, we collected gastric cancer tissues from 89 patients and constructed a tissue microarray for immunohistochemical detection of c-Met and YAP expression. Our findings revealed that both Met and YAP were significantly upregulated in gastric cancer tissues compared with paraneoplastic tissues (*P* < 0.001), while high expression levels of these proteins were associated with shortened survival times (*P* < 0.01). Besides, to further expand the data queue and diversity, we downloaded patient survival data from TCGA and KMPLOT analysis, and the results also showed the high expression of Met and YAP in gastric cancer tissues, and the inverse relationship between their overexpression and prognosis. These results are consistent with those previously reported [21, 22].

Through multiple correlation analyses, both microarray and bioinformatics methods, we found that Met was significantly correlated with YAP. Further *χ*2 test revealed a significant positive correlation between Met expressions and P53 as well as CD133 (*P* < 0.05). YAP expressions were found to be positively associated with CD133 (*P* < 0.05). Pearson's correlation analysis further demonstrated a distinct relationship between Met expression and VEGFR as well as CD133 (*P* < 0.01), while YAP expression was correlated with Ki67 and VEGFR expression (*P* < 0.05). These results all showed a strong binding of Met and YAP. But further COX regression analysis found the Met expression, N stage, and VEGFR expression as independent risk factors for gastric cancer (*P* < 0.05), whereas YAP expression was not significant (*P* > 0.05). Met exhibits greater prominence in gastric cancer compared to YAP. Given its close association with carcinogenesis and poor prognosis, Met may serve as a potential prognostic molecular marker or therapeutic target for gastric cancer.

However, some scholars argue that YAP exerts an upstream influence on tumors in comparison to Met. Yan et al. [29] reported that the activation of YAP drives the transcription of c-Met, thereby promoting the expression of c-Met; Thomann et al. [27] discovered that YAP regulates heterotypic communication among endothelial cells in liver tumorigenesis through HGF/c-MET signaling. Hence, further investigation is required to establish the causal relationship between Met and YAP in gastric cancer.

## 5. Limitations

We only collected tissues from 89 patients, the generalizability of the findings may be limited, and the validity could be enhanced by including a larger, more diverse patient cohort. Other than the clinical outcomes above, more potential confounders should be further addressed to increase the credibility of the results.

## 6. Conclusion

The high expressions of Met and YAP exhibit a strong correlation with the clinicopathological features and overall survival of patients diagnosed with gastric cancer. Met, as an independent risk factor, significantly influences the prognosis of gastric cancer and holds potential as a prognostic molecular marker or therapeutic target.

## Figures and Tables

**Table 1:** Correlation between Met, YAP expressions, and clinicopathological features.

	Variables	Met expression		*χ*2	*P* value	YAP expression		*χ*2	*P* value
		Low	High			Low	High		
Age (year)	<=65	18	26	0.786	0.375	24	11	2.764	0.096
	>65	14	30			22	22		

Sex	Female	7	23	3.131	0.077	16	12	0.046	0.830
	Male	25	34			31	21		

Grade	I-II	7	7	1.423	0.233	11	2	4.285	0.038
	III-IV	25	50			36	31		

T stage	II	6	6	0.538	0.463	8	2	1.245	0.264
	III-IV	26	50			39	31		

N stage	N0	7	15	3.287	0.349	11	8	1.173	0.760
	N1	8	6			10	4		
	N2	7	16			12	10		
	N3	10	20			14	11		

TNM stage	I-II	15	18	2.055	0.152	21	10	1.689	0.194
	III-IV	17	39			26	23		

P53	Low	18	22	3.981	0.046	20	14	0.009	0.926
	High	11	34			26	19		

Ki67	Low	14	22	0.632	0.426	18	12	0.062	0.803
	High	15	34			28	21		

VEGFR	Low	14	23	0.403	0.525	20	14	0.009	0.926
	High	15	33			26	19		

CD133	Low	22	18	14.658	<0.001	25	10	4.502	0.034
	High	7	38			21	23		

E-Cadherin	Low	18	24	2.298	0.130	23	18	0.159	0.690
	High	12	32			23	15		

**Table 2:** Correlation analysis of Met, YAP, and tumor-related proteins.

	Met		YAP	
	*R*	*P*	*R*	*P*
P53	0.185	0.090	0.095	0.403
Ki67	0.153	0.162	0.250	0.027
VEGFR	0.368	0.001	0.285	0.011
CD133	0.385	<0.001	0.225	0.046
E-Cadherin	0.163	0.135	0.067	0.555

**Table 3:** The Cox multivariate regression analysis of gastric cancer survival.

Variables	Univariate analysis			Multivariate analysis		
	HR	95% CI	*P* value	HR	95% CI	*P* value
Met expression	2.286	1.267-4.127	0.006	1.865	1.019-3.411	0.043
YAP expression	2.005	1.173-3.430	0.011	1.293	0.651-2.567	0.463
Sex	0.817	0.500-1.335	0.420			
Grade	2.398	1.034-5.562	0.042	2.156	0.820-5.674	0.120
Age	1.887	1.147-3.103	0.012	1.572	0.836-2.957	0.160
T stage	2.556	1.024-6.379	0.044	1.345	0.494-3.663	0.562
N stage	1.394	1.122-1.732	0.003	1.839	1.109-3.047	0.018
TNM stage	2.284	1.353-3.857	0.002	0.704	.218-2.273	0.557
P53	1.252	0.771-2.033	0.364			
Ki67	0.679	0.417-1.104	0.119			
VEGFR	1.680	1.029-2.743	0.038	2.311	1.280-4.175	0.005
CD133	1.360	0.839-2.205	0.212			
E-Cadherin	0.742	0.457-1.204	0.227			

## Data Availability

The data is available from the corresponding authors, upon reasonable request.
